# The effect of left ventricular contractility on arterial hemodynamics: A model-based investigation

**DOI:** 10.1371/journal.pone.0255561

**Published:** 2021-08-02

**Authors:** Stamatia Pagoulatou, Dionysios Adamopoulos, Georgios Rovas, Vasiliki Bikia, Nikolaos Stergiopulos

**Affiliations:** 1 Laboratory of Hemodynamics and Cardiovascular Technology (LHTC), Institute of Bioengineering, Ecole Polytechnique Fédérale de Lausanne (EPFL), Lausanne, Switzerland; 2 Cardiology Department, Geneva University Hospitals (HUG), Geneva, Switzerland; University of Ljubljana, Medical faculty, SLOVENIA

## Abstract

Ventricular-arterial coupling is a major determinant of cardiovascular performance, however, there are still inherent difficulties in distinguishing ventricular from vascular effects on arterial pulse phenotypes. In the present study, we employed an extensive mathematical model of the cardiovascular system to investigate how sole changes in cardiac contractility might affect hemodynamics. We simulated two physiologically relevant cases of high and low contractility by altering the end-systolic elastance, *E*_*es*_, (3 versus 1 mmHg/mL) under constant cardiac output and afterload, and subsequently performed pulse wave analysis and wave separation. The aortic forward pressure wave component was steeper for high *E*_*es*_, which led to the change of the total pressure waveform from the characteristic *Type A* phenotype to *Type C*, and the decrease in augmentation index, *AIx* (-2.4% versus +18.1%). Additionally, the increase in *E*_*es*_ caused the pulse pressure amplification from the aorta to the radial artery to rise drastically (1.86 versus 1.39). Our results show that an increase in cardiac contractility alone, with no concomitant change in arterial properties, alters the shape of the forward pressure wave, which, consequently, changes central and peripheral pulse phenotypes. Indices based on the pressure waveform, like *AIx*, cannot be assumed to reflect only arterial properties.

## Introduction

The arterial blood pressure is the result of the instantaneous interaction between the left ventricle (LV) of the heart and the arterial system. The ventricular-arterial coupling is a major determinant of left ventricular function and global cardiovascular health [1–3]. Accordingly, several physiological and pathological processes are linked with deleterious alterations in one component of the interaction, which gradually compromise the function and structure of its counterpart. Ageing, for example, is linked with the stiffening of the arterial tree [4, 5]; the increase in vascular load triggers remodeling of the LV, which leads to further increases in systemic pressure and so on [6].

Distinguishing myocardial from vascular effects is critical to deciphering the interaction between the two systems and assessing cardiovascular performance. Previous clinical-epidemiological studies have undertaken this task by investigating arterial hemodynamics in normal and diseased human hearts under varying loading conditions and inotropic states [1, 7–9]. Particularly, different states of cardiac contractility are often assessed via the use of medication that increases heart rate and cardiac output [7, 8].

However, there are inherent difficulties in studying *in vivo* the hemodynamic effect of purely inotropic changes. First, such studies require invasive techniques to measure intraventricular pressures and volumes, which pose significant risks and are applicable to only special settings. Second, it is practically impossible to isolate myocardial effects, as the agents used influence also the vascular system, often altering mean arterial pressure [7, 8]. To overcome this limitation, physiology-based models of the cardiovascular system can be of use, as they allow for changes of cardiac parameters while keeping the arterial system unchanged.

Over the past decades, a number of lumped- and distributed-parameter mathematical models of the cardiovascular system have been developed and used in order to provide insights into circulatory physiology and pathology [10–14]. Overall, zero-dimensional (0D) models are suitable for the simulation of global hemodynamics as well as the study of interactions between modelled components; however, they lack spatial dimension. Contrarily, one-dimensional (1D) models of the vasculature, which are based on a simplified version of the 1D form of the Navier-Stokes equations, are regarded as a reliable and convenient tool for investigating wave transmission phenomena in the cardiovascular system. One of the most complete and accurate 1D cardiovascular models described in the current literature was developed and validated in our laboratory by Reymond et al. [12, 15]. In the present study, we employed our state-of-the-art, 1D model of the cardiovascular system to investigate how sole changes in cardiac contractility might affect central and peripheral hemodynamics. To that aim, we simulated two physiologically relevant cases of high and low LV contractility under constant cardiac output and afterload. Particularly, we analyzed the effect of changes in cardiac contractility on key features of the aortic and radial pressure and flow waves, wave propagation characteristics, as well as the pulse pressure amplification from the ascending aorta to the radial artery.

## Materials and methods

### Brief description of the model of the cardiovascular system

The mathematical model [12] solves the one-dimensional Navier-Stokes equations combined with a constitutive law for the wall elasticity along the centerline of each artery. The arterial tree consists of a network of 103 arteries, including 55 main systemic arteries, the coronary circulation and a representation of the circle of Willis. Proximally, the ascending aorta is coupled with a 0D model of the left ventricle based on the varying elastance model [16, 17]. Importantly, the contractility of the LV is described by the linear end-systolic pressure-volume relationship (*ESPVR*) and particularly its slope, the end-systolic elastance (*E*_*es*_), which is sensitive enough to detect pathologies such as hypertrophic and dilated cardiomyopathies [18].

The complete mathematical model was proposed by Reymond et al. in 2009 [12] and was thoroughly validated against *in vivo* data [15]. Over the past years, the original model has been improved to include a better description of the valve dynamics [19]. Additionally, the intraventricular pressure-volume relation has been updated to consider the non-linearity of the end-diastolic pressure-volume relationship (*EDPVR*) [20]. In the present work, the LV pressure (*P*_*LV*_) is the addition of a linear *ESPVR* and an exponential *EDPVR*. The contraction is modulated by a time-varying activation function, *ϵ*(*t*), which varies from 0 to 1 and controls the weights of the two terms as follows:

$$P_{LV}(V_{LV})=\epsilon (t)*ESPVR+(1-\epsilon (t))*EDPVR$$

Where *ESPVR* is equal to *E*_es_*(*V*_*LV*_−*V*_*d*_), with *E*_*es*_ being the end-systolic elastance and *V*_*d*_ the dead volume [17], and *EDPVR* is equal to *P*_0_*exp(β*V_*LV*_), with *P*_0_ being the dead pressure and *β* a diastolic stiffness parameter. The time-varying activation is modelled by the double-hill function proposed by Stergiopulos et al. [21].

A detailed description of the model equations is provided in S1 Appendix.

### Setup of modelling parameters

In this work, we used the cardiovascular model described above to run two discrete simulations. The first simulation pertained to a virtual healthy subject who had high LV contractility and an arterial tree of physiological parameters, representative of a middle-aged male. The second simulation corresponded to a different virtual healthy subject who shared the exact same arterial tree with the first one, but had lower LV contractility.

The cardiovascular parameters of these two subjects were chosen according to the physiological ranges and mechanisms reported in the literature (Table 1). More specifically, for the first subject we imposed a steep *ESPVR*, with maximal end-systolic elastance *E*_*es*_ = 3 mmHg/mL. This value is in the upper limit of the physiological range reported in the publication by Senzaki et al. [18] for normal hearts. The dead volume, *V*_*d*_, was set at -2 mL, which is in the normal range of [–100, 100] mL [22, 23]. For this high contractility scenario, the LV preload (i.e., the end-diastolic pressure, EDP) was set at 8.2 mmHg [24] and the end-diastolic volume (EDV) at 97 mL [25]. The simulation yielded a physiologic stroke volume (SV) of 65mL, with a high-normal EF of 68% [25].

**Table 1:** Model parameters used to set up the two scenarios of high and low LV contractility.

Parameters	High contractility Simulation	Low contractility Simulation
**LV parameters**		
*ESPVR*		
End-systolic elastance, *E*_*es*_ (mmHg/mL)	3.0	1.0
Dead volume, *V*_*d*_ (mL)	-2	-60
Ejection Fraction, EF	68%	54%
*EDPVR*		
Preload, EDP (mmHg)	8.2	11.0
End-diastolic volume, EDV (mL)	97	120
Dead pressure, *P*_0_ (mmHg)	2.3	
Diastolic stiffness, *β* (mL^-1^)	0.013	
Heart rate, HR (bpm)	70	
**Arterial Parameters**		
Arterial compliance, *C*_*A*_ (mL/mmHg)	0.62	
Terminal compliance, *C*_*T*_ (mL/mmHg)	0.26	
Total vascular compliance, *TVC* (mL/mmHg)	0.88	
Total vascular resistance, *TVR* (mmHg‧sec/mL)	1.22	
Aortic Characteristic Impedance, *Z*_*c*_ (mmHg‧sec/mL)	0.04	

**Fig 1:**
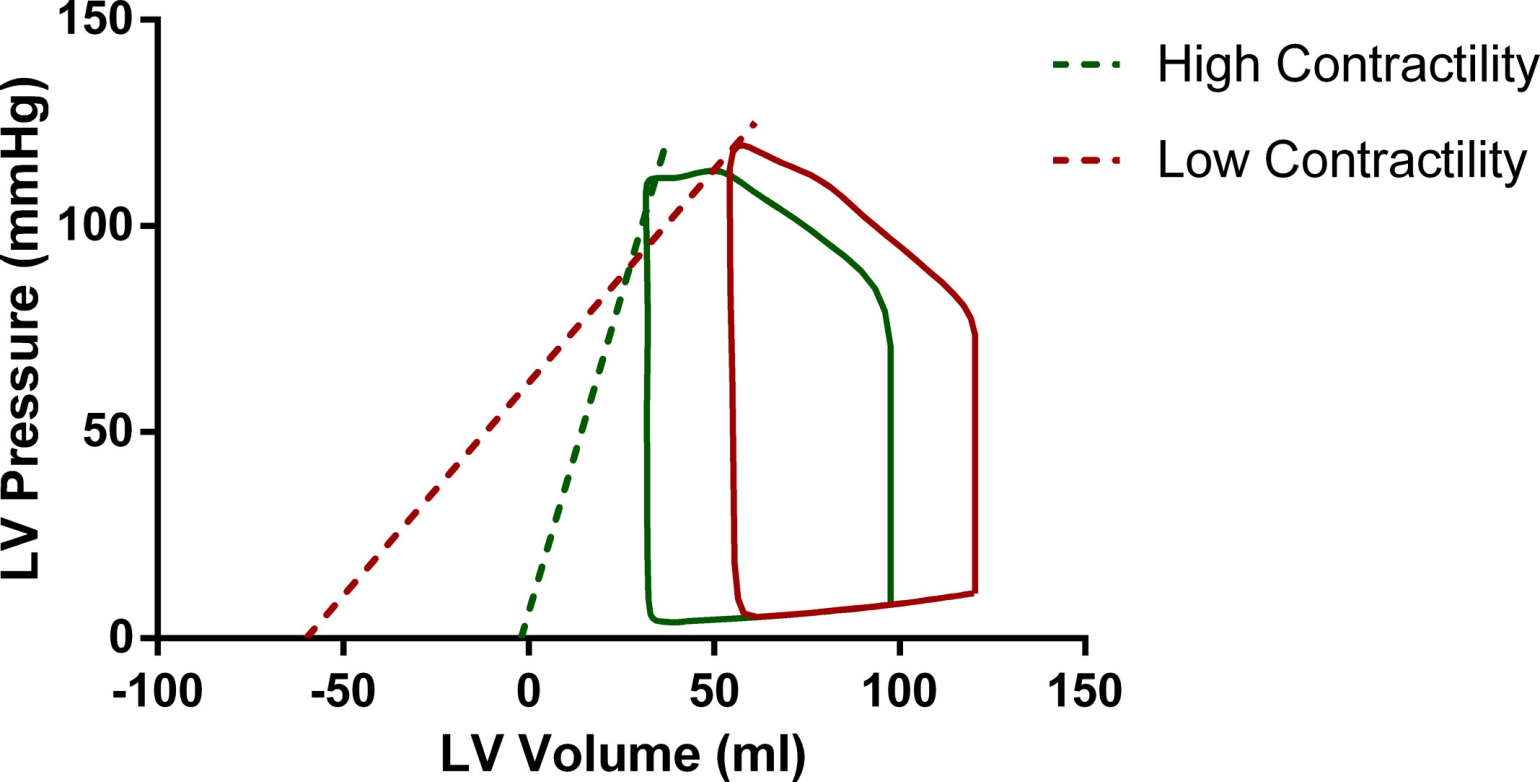
Intraventricular pressure-volume loops for two scenarios of high (*E*_*es*_ = 3 mmHg/mL, *V*_*d*_ = −2 mL—green curve) and low (*E*_*es*_ = 1 mmHg/mL, *V*_*d*_ = −60 mL—red curve) LV contractility with a maintained stroke volume. Note that for the low *E*_*es*_ scenario an increase in preload was needed in order to restore stroke volume. The dashed lines represent the respective linear ESPVRs.

For the second subject, we introduced a decrease in LV contractility. Concretely, we set *E*_*es*_ = 1 mmHg/mL, which is in the lower limit of the range proposed for normal hearts [18], and *V*_*d*_ = −60 mL. In absence of compensatory mechanisms, this decrease of *E*_*es*_ would lead to a drop in the stroke volume. The physiological mechanism to restore the SV is through increase of the preload as explained by the Frank-Starling law. Therefore, in our simulation, in order to maintain the stroke volume constant for both subjects, SV = 65 mL, the preload had to increase by 2.8 mmHg, i.e. from 8.2 to 11mmHg. The increase of preload was accompanied by a mild dilation of the LV, i.e. EDV increased to 120 mL and EF dropped to 54%. Both changes in the EDV and EF are still in the physiological range [25]. Fig 1 demonstrates the resulting intraventricular P-V loops for the high and low contractility scenarios. These two scenarios will be hereafter denoted as *E*_*es*_ ↑ for high and *E*_*es*_ ↓ for low contractility.

The diastolic properties of the LV were set at normal values [26] and were equal for both simulations (Table 1). To isolate the cardiac effects, we employed for both cases the same arterial tree model, which had physiologically relevant parameters representative of a healthy middle-aged adult [27] (Table 1). Normal valve properties were chosen according to [19]. Cardiac and arterial parameters for the two simulations can be found in Table 1.

### Data analysis and wave separation

**Fig 2:**
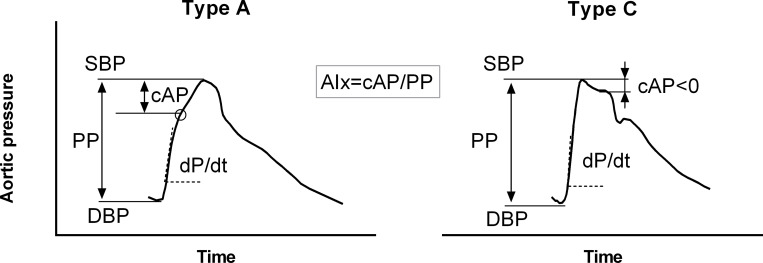
Aortic pulse wave analysis for the characteristic Type A and Type C phenotypes. SBP: systolic blood pressure, PP: pulse pressure, DBP: diastolic blood pressure, cAP: central augmentation pressure, AIx: augmentation index.

From the results of the two simulations, key features of the flow and pressure waveforms were extracted including: the magnitude and timing of the peak of the aortic flow, the aortic and radial systolic (*SBP*), diastolic (*DBP*) and pulse (*PP*) pressure, the maximal slope of the aortic pressure upstroke (*dP*/*dt*_*max*_). The pulse pressure amplification (*PP*_*amp*_) between the proximal aorta and the radial artery was calculated as the ratio *PP*_*radial*_/*PP*_*aortic*_ [28]. Central (aortic) Augmentation Pressure (*cAP*) was defined according to the characteristic inflection point or “shoulder” on the aortic pressure waveform. Accordingly, central Augmentation Index (*cAIx*) was expressed as the ratio of central augmented pressure to the central pulse pressure *cAP*/*cPP* [29]. The two aortic pressure waveforms were also classified into 2 types according to the timing of the inflection point as previously described by Murgo et al. [29]: the *Type A* pressure waveform, whereby the peak systolic pressure occurs after the shoulder and *cAIx*>12%, and the *Type C* pressure waveform, whereby the peak systolic pressure precedes the inflection point and *cAIx*<0 (Fig 2). Peripheral (radial) Augmentation Index (*pAIx*) was the ratio of the amplitude of the late systolic peak to the amplitude of the early systolic peak [30].

Subsequently, aortic pressure waveforms were separated into their forward and backward traveling components as in Westerhof et al. [31]. Aortic characteristic impedance was calculated by averaging the modulus of the input impedance in the frequency range between 3–9 harmonics [31]. The amplitude, peak and upstroke steepness for the forward and backward pressure waves were quantified. Reflection coefficient was defined as the ratio of backward wave amplitude over forward wave amplitude.

## Results

**Fig 3:**
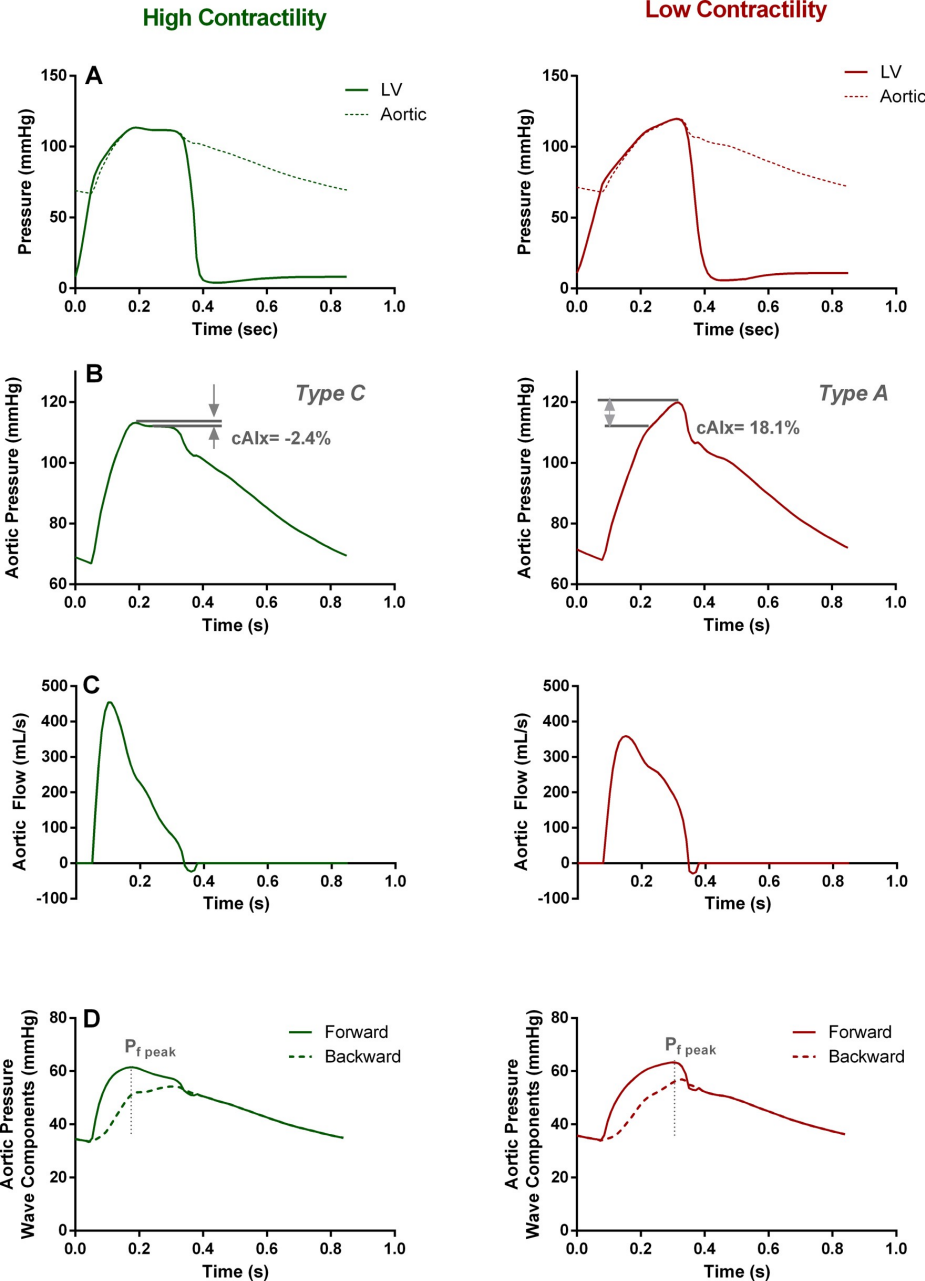
Effect of LV contractility on central hemodynamics. (**A)** LV and aortic pressure as a function of time. **(B)** Characteristic *Type C* and *Type A* aortic pressure phenotypes reproduced by high and low contractility, respectively. **(C)** Aortic flow. **(D)** Forward and backward travelling pressure waves. Note that the forward peak occurs significantly earlier in the high contractility simulation, while the amplitudes of the forward and backward pressure waves are left unchanged.

**Fig 4:**
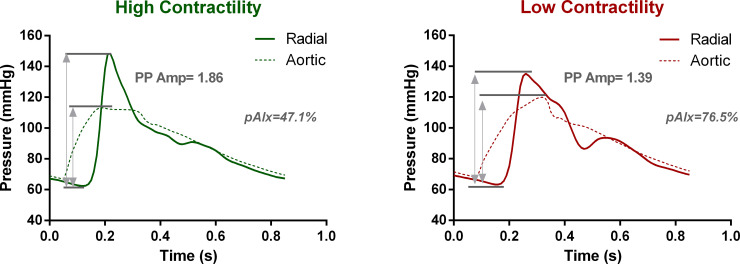
Central and peripheral arterial pressure for the two cases of LV contractility. Note the rise in the radial systolic pressure as well as in the pulse pressure amplification when *E*_*es*_ is higher; in this case, *pAIx* is significantly lower.

The main pressure and flow characteristics for the increased and decreased contractility simulated cases are presented in Table 2 as well as in Figs 3 and 4. We find that changes in the *ESPVR* have a major impact on both central and peripheral hemodynamics. For *E*_*es*_ ↑ the LV pressure curve has a steep upstroke (14.9‧10^2^ mmHg/sec), reaches its peak early in systole (0.14 sec after beginning of ejection) and then slowly decreases until the end of systole (Fig 3A *left* and Table 2). Contrarily, for *E*_*es*_ ↓ the slope of the LV pressure is almost halved (8.7‧10^2^ mmHg/sec), the aortic pressure meets the LV curve towards the end of systole, when the LV pressure peak occurs (0.23 sec after beginning of ejection) (Fig 3A *right* and Table 2). It is of interest to observe that the maximal LV pressure is lower in the case of high contractility (113.6 mmHg for *E*_*es*_ ↑ versus 119.7 mmHg for *E*_*es*_ ↓). These features can also be observed on the P-V loops depicted in Fig 1.

**Table 2:** Comparison of hemodynamic characteristics between the two contractility simulations.

Parameters	High Contractility Simulation	Low Contractility Simulation
*Aortic flow*		
Cardiac Output (L/min) (maintained)	4.6	4.6
Maximal Aortic Flow (mL/sec)	453	359
Timing of Maximal Aortic Flow (sec) (since beginning of ejection)	0.06	0.07
Maximal slope, dF/dt max (mL/sec^2^)	1.53e4	1.09e4
*LV P-V*		
Maximal *P*_*LV*_ (mmHg)	113.6	119.7
Timing of maximal *P*_*LV*_ (sec) (since beginning of ejection)	0.14	0.23
LV dP/dt max (mmHg/sec)	14.9e2	8.7e2
*Arterial pressure*		
Aortic SBP (mmHg)	113.2	119.6
Aortic DBP (mmHg)	66.9	67.9
Aortic MAP (mmHg)	91.1	92.0
Aortic PP (mmHg)	46.3	51.8
Aortic AP (mmHg)	-1.0	9.4
Aortic AIx (%)	-2.4	18.1
Aortic dP/dt max (mmHg/sec)	6.6e2	4.6e2
Radial SBP (mmHg)	148.3	135.1
Radial DBP (mmHg)	62.4	63.3
Radial MAP (mmHg)	87.4	88.3
Radial PP (mmHg)	85.9	71.9
Radial AIx (%)	47.1	76.5
PP Amplification	1.86	1.39
*Wave Separation Analysis*		
Forward pressure wave component amplitude (mmHg)	28.1	29.5
Forward pressure wave peak (mmHg)	61.5	63.3
Forward pressure wave peak timing (sec) (since beginning of ejection)	0.12	0.23
Maximal slope forward dP/dt (mmHg/sec)	6.6e2	4.4e2
Backward pressure wave component amplitude (mmHg)	20.7	22.7
Backward pressure wave peak (mmHg)	54.3	56.9
Backward pressure wave peak timing (sec) (since beginning of ejection)	0.25	0.25
Reflection coefficient (%)	73.7	76.9

SBP: Systolic Blood Pressure, DBP: Diastolic Blood Pressure, MAP: Mean Arterial Pressure, PP: Pulse Pressure, AP: Augmentation Pressure, AIx: Augmentation Index.

Accordingly, for increased *E*_*es*_ both aortic flow and pressure curves have steeper upstrokes at the beginning of ejection and reach their respective peaks earlier (Fig 3B and 3C and Table 2). Importantly, we note that the aortic flow wave shape is distinctively different between the two cases. Even though cardiac output is preserved, the maximal flow value is significantly higher for *E*_*es*_ ↑ (453 mL/sec for *E*_*es*_ ↑ versus 359 mL/sec for *E*_*es*_ ↓) (Fig 3C and Table 2). Additionally, we observe differences in the shape of the aortic pressure waves: although both simulations refer to the same total compliance and the same characteristic impedance of the proximal aorta (Table 1), the aortic pressure waveform resembles the characteristic *Type C* phenotype for *E*_*es*_ ↑, while for *E*_*es*_ ↓ the curve resembles the *Type A* phenotype (Fig 3B). Naturally, this is also reflected on the central AIx, which rises from -2.4% for *E*_*es*_ ↑ to +18.1% for *E*_*es*_ ↓.

Fig 3D contains an overview of the results of the wave separation analysis. For both cases, the amplitudes of the aortic forward and backward pressure wave components are approximately the same, which leads to the preservation of their ratio, i.e. the same reflection coefficient (Table 2). Overall, the backward wave seems relatively unaffected by the change in contractility. However, this does not hold true for the shape of the forward wave. Concretely, the peak of the forward pressure wave is pushed earlier in systole when LV contractility is increased (0.12 sec for *E*_*es*_ ↑ versus 0.23 sec for *E*_*es*_ ↓), which entails the increase of the steepness of its upstroke (6.6‧10^2^ mmHg/sec for *E*_*es*_ ↑ versus 4.4‧10^2^ mmHg/sec for *E*_*es*_ ↓). This finding is in line with previous observations [32, 33].

The effect of *ESPVR* is not limited to only central hemodynamics. In Fig 4, we demonstrate the pressure waveforms at the distal radial artery as predicted by the model for the high and low *E*_*es*_ values. The radial mean pressure is rather conserved (Table 2). However, we note that an increase in *E*_*es*_ leads to a pronounced increase in radial SBP (148.3 mmHg for *E*_*es*_ ↑ versus 135.1 mmHg for *E*_*es*_ ↓) and radial PP (85.9 mmHg for *E*_*es*_ ↑ versus 71.9 mmHg for *E*_*es*_ ↓) (Table 2). Evidently, this causes the pulse pressure amplification from the ascending aorta to the radial artery to rise drastically, i.e. *PP*_*amp*_ is 1.86 for *E*_*es*_ ↑ versus 1.39 for *E*_*es*_ ↓. With respect to the shape of the radial pressure curve, the late systolic peak has similar timing for both cases, however, its value is lower for *E*_*es*_ ↑. This is translated in a drop in the peripheral augmentation index, i.e. *pAIx* is 47.1% for *E*_*es*_ ↑ vs 76.5% for *E*_*es*_ ↓.

## Discussion

In the present study, we demonstrated that an increase in LV contractility alone could directly result in alterations in both central and peripheral hemodynamics even for an unchanged arterial load and cardiac output. This was achieved by employing an extensive, physiologically relevant mathematical model of the cardiovascular system, and manipulating the end-systolic pressure-volume relation in order to simulate higher and lower systolic function. This work addresses the hemodynamic footprint of contractility on multiple levels, touching upon its effect on the central pressure and flow waveforms as well as the distal radial pressure phenotypes.

Importantly, we found that an increase in LV contractility has an effect on the shape of the initial forward travelling wave pumped by the left ventricle; the forward wave shows a pronounced upstroke and an early peak, without, however, changing its amplitude (Fig 3D). On the other hand, the wave reflections are not particularly affected, as they depend primarily on vascular properties (Fig 3D).

The increased steepness of the forward wave due to increased contractility orchestrates a number of changes in both central and peripheral arterial hemodynamics. The respective proximal aortic pressure and flow waveforms change drastically in shape, i.e. they become steeper, reach their peak values earlier in systole, and particularly for the aortic waveform the peak value is significantly increased. Of interest is the fact that when *E*_*es*_ is decreased the aortic pressure curve resembles the characteristic *Type A* phenotype, while for increased *E*_*es*_ it resembles the *Type C* phenotype. Accordingly, we note alterations in the aortic inflection point and AIx; for increased LV contractility the AIx drops and might even become negative (Fig 3B and Table 2).

In addition to central hemodynamics, changes in cardiac contractility affect also peripheral hemodynamic phenotypes. Radial systolic and pulse pressure increases for higher *E*_*es*_, although the mean pressure is preserved (Fig 4 and Table 2). This finding might seem rather counter-intuitive at first. Since we employ the same arterial tree for both simulations, the transmission line theory dictates that there should be no change in the transmission/reflection coefficients from the central aorta to the radial artery. Indeed, the reflection coefficient calculated as the ratio of the backward to forward wave amplitude remains constant. Therefore, one would expect that the pulse pressure amplification should also be maintained despite the changes in LV contractility. This disparity can be explained by the effect of *E*_*es*_ on the slope/timing of forward wave. In other words, even though the transmission network is not altered, the initial forward wave pumped by the heart is. The fact that the forward wave is characteristically steeper and reaches its peak early suggests that at a specific time point in early systole, the forward pressure wave has a higher value, which will result in an also amplified radial pressure (Fig 3).

In light of this evidence, we can better appreciate observations made in previous clinical works [34, 35]. Particularly, we recently investigated the hemodynamic profile of patients with severe aortic valve stenosis (AS) before and acutely after they underwent Transcatheter Aortic Valve Replacement (TAVR) [25]. Interestingly, we found significant differences between the shape of central pressure and flow waves before and after TAVR. We showed that resolution of aortic stenosis led to an enhanced forward traveling wave, which was associated with changes in the central pressure and flow waveforms as well as a decrease in the aortic AIx. We hypothesized that post-TAVR hemodynamics might be related to the hyperdynamic state of the LV, which cannot acutely adapt to the improved loading conditions. The present work supports this hypothesis and offers a mathematical explanation of this clinical observation.

### Clinical implications

Our findings have several implications. First, central as well as peripheral pressure and flow waveforms might contain crucial information on cardiac systolic function. This point has also been evoked in previous studies that proposed [9, 36] and used [34, 37, 38] aortic dP/dt_max_ as a measure of LV contractility. A recent clinical study by [9] suggested that this does not apply only to central waveforms but also extends to peripheral measures, i.e. radial and femoral dP/dt_max_ were also able to track reasonably well LV inotropic changes. Here, we provide a mathematical justification of why this holds true: LV contractility affects primarily the forward wave and it is in fact the slope of the forward wave that is captured in both central and peripheral dP/dt_max_ measures.

Further advancing this line of thinking, we suggest that the forward wave might be an important element of the ventricular-arterial coupling [39]; indeed, its slope and the timing of its peak seem to be informative of the LV systolic function. Contrarily, the total backward travelling wave is rather a vascular index and provides information on the cumulative effect of reflections occurring throughout the arterial tree. Even though wave separation analysis is not currently being performed as part of the clinical routine, it can potentially offer a complete image of the cardiovascular coupling and assist clinicians with better assessing LV performance.

A second finding of major clinical importance is that high peripheral SBP might not necessarily be indicative of central systolic hypertension. Aortic pressure, which is directly “seen” by the heart, strongly relates to vascular disease and clinical outcomes [40]. However, since access to aortic pressure requires invasive measurements, peripheral pressure measurements are used instead. Here, we demonstrate that PP amplification from the aorta to the radial artery changes with changes in *E*_*es*_, which partly explains previous doubts expressed on the reliability of peripheral pressure as a surrogate of central pressure [40–42]. Arguably, peripheral pressure is a well-studied marker with prognostic value for cardiovascular morbidity and mortality [43]. Nevertheless, we suggest that it might be meaningful to focus more attention on deriving central pressure as it offers a better description of the afterload.

On that note, one should also exert caution when interpreting the shape of the central pressure waveform to assess vascular stiffness. In the present study, we were able to reproduce both *Type A* and *Type C* aortic pressure phenotypes [29] by changing only the cardiac systolic properties, while maintaining the arterial tree parameters of an average middle-aged male. Traditionally, *Type A* pressure curves are associated with increased aortic stiffness and advanced age, whereby large reflections arrive earlier back to the aortic root. Contrarily, *Type C* pressure curves are understood to represent younger individuals, with lower amplitude reflections and lower pulse pressures [29]. Here, we demonstrate that these particular phenotypes are not solely dictated by vascular stiffness and reflections, but are actually strongly related to cardiac contractility. This comment also extends to the central AIx, which was until recently regarded as a purely vascular parameter [44]. We show that cardiac contractility is also an important determinant of central AIx, a finding that corroborates previous statements that AIx might not be a measure of wave reflections solely [32, 45, 46].

### Considerations on the analysis and study limitations

When interpreting our results the reader should consider that the data presented pertain to mathematical simulations and not *in vivo* human measurements. Nevertheless, this limitation is mitigated by the facts that: i) the mathematical model used in the present study has been thoroughly validated against *in vivo* data before and has been found capable of accurately representing physiologic and pathologic hemodynamics [15, 27], ii) the model parameters were chosen in a way to reflect plausible physiological states, according to normal ranges proposed in the literature (the choice of each model parameter was respectively justified), iii) our findings match well previously reported data from clinical and modeling studies [32, 33, 45].

On that note, we should acknowledge that we do not have complete understanding on how changes in LV contractility will exactly affect the volume intercept, *V*_*d*_, of the *ESPVR*. Arguably, we anticipate that when contractility decreases, the preload should increase due to the Frank-Starling law, thus reducing the EF; however, this change in preload and EF largely depends on the chosen *V*_*d*_ values. Here, we chose the *V*_*d*_ values, so that a drastic change in LV contractility would lead to an equally drastic change in EF, i.e. when *E*_*es*_ dropped from 3 mmHg/mL to 1 mmHg/mL, EF also decreased from 68% to 54%. In an exaggerated version, this mechanism describes systolic heart failure: when LV contractility is compromised, the increase in preload does no longer suffice to restore the stroke volume and EF gradually drops to pathologic values.

Additionally, it should be noted that our observations regarding the alteration of central and peripheral wave characteristics due to changes in LV contractility are not sensitive to afterload and preload, as demonstrated in S2 Appendix. More specifically, we showed that repeating the analysis for an unchanged preload (i.e., LVEDP = 11mmHg, for both high and low contractility cases would only minimally affect the simulated central and peripheral pressure waves (S2.1 Table in S2 Appendix). Similarly, the major study conclusions would still hold true after altering the afterload, i.e. changing the aortic compliance and peripheral resistance by ±20% (S2.2, S2.3 Table in S2 Appendix).

The cardiac model of time-varying elastance implemented here is based on the assumption that the normalized elastance, *E*_*N*_(*t*), shares a uniform shape among different individuals [17]. This view has been challenged as previous studies showed a significant variation of *E*_*N*_(*t*) according to afterload and introduced correction models [47]. This feature is not yet included in our simulations, but will be incorporated in our future studies. However, since vascular load was kept constant in the present study we do not expect that it would significantly affect our results.

Additionally to the concept of elastance, other mathematical models have been proposed to describe the contractility of the LV. Particularly, more detailed finite-element models exist [48, 49] that couple cavity mechanics with sarcomere mechanics. The use of such a model (like the one proposed by [48]) might be more relevant for the investigation of hemodynamic changes under varying inotropic states. Future work will be oriented towards this direction. Further model-related limitations can be found in the original publication [12].

## Conclusions

In the present study, we demonstrated by means of a mathematical model of the cardiovascular system that a physiological increase in cardiac contractility leads to a steeper forward pressure wave pumped by the LV, which, subsequently, drastically alters central and peripheral pressure and flow waves. This might have important implications for the assessment of cardiac contractility through measurement of noninvasive pressure waveforms. Additionally, the characteristic *Type A* and *Type C* aortic pressure phenotypes, and accordingly the central AIx, are not solely dictated by vascular stiffness but also majorly depend on LV contractility. Last, we found that the amplification of the pulse pressure from the central aorta to the periphery is also affected by the cardiac contractile state and, hence, suggest that caution should be exerted when using peripheral measurements as surrogate for central pressure.

## Supporting information

S1 Appendix Description of the mathematical model of the cardiovascular system. (DOCX)

S2 Appendix Sensitivity analysis. (DOCX)
