# Supplementary material for: The effect of left ventricular contractility on arterial hemodynamics: A model-based investigation
Source: PLoS One. 2021 Aug 2;16(8):e0255561. doi: 10.1371/journal.pone.0255561 (PMC8328319; doi:10.1371/journal.pone.0255561)
Supplement: S1 Appendix — (DOCX) [file pone.0255561.s001.docx]

**S1 Appendix: Description of the Mathematical Model of the Cardiovascular System**

This mathematical model is based on the original work of Reymond et al. [1], which has been updated to include valve dynamics and a 0D model of the left ventricle (LV) of the heart, where the non-linear diastolic LV properties are described. The following paragraphs offer an overview of the model and detail on the governing equations. Additionally, Supplementary Figure S1.1 offers a schematic representation of the complete model.

LV Contractility Model

The LV is modeled as a pump, which feeds from a constant pressure reservoir (i.e., modelled as a pressure source and a resistance) and pressurizes blood into the aorta. The time-varying contraction model of the LV is based on the concept of elastance [2]. More specifically, LV pressure-volume relation, $P_{LV}$ ($V_{LV})$, is defined by a linear end-systolic pressure-volume relation ($ESPVR)$ [3] and an exponential end-diastolic pressure-volume relation ($EDPVR$) [4], where:

$ESPVR=E_{es}*(V_{LV}-V_{d})$ (1)

$EDPVR=P_{0}*exp(\beta*V_{LV})$ (2)

With $E_{es}$ being the end-systolic elastance and $V_{d}$ the dead volume [3], $P_{0}$ the dead pressure and $\beta$ a diastolic stiffness parameter.

The contraction/relaxation is modulated by a time-varying activation function,$\epsilon\left( t \right),$ which varies from 0 to 1 and controls the weights of the ESPVR and EDPVR terms as follows:

$P_{LV}$ ($V_{LV})$ =$\epsilon\left( t \right)*ESPVR+\left( 1-\epsilon\left( t \right) \right)*EDPVR$ (3)

In the present work, the time-varying activation is modelled using the normalized double-hill function proposed by Stergiopulos et al. [5].

Aortic valve model

The pressure drop ($\Delta p$) across the aortic valve is modelled based on the work of Mynard et al. [6], where losses related to turbulence and inertial acceleration of the blood are incorporated:

$$\Delta p=BQ\left| Q \right|+L\frac{dQ}{dt} (4)$$

with Q being the flow through the valve. Coefficients B and L reflect the relative contribution of the turbulence and inertia effects to the pressure drop, respectively, and depend on blood density ($\rho$), the effective valve area ($A_{eff}$), and valve length ($l$):

$$B=\frac{\rho}{2{A_{eff}}^{2}} (5)$$

$$L=\rho\frac{l}{{A_{eff}}} (6)$$

The effective valve area varies between a minimal ($A_{min})$ and maximal ($A_{max}$) value via a valve opening/closing function, $\zeta(t)$:

$$A_{eff}=\left( A_{max}-A_{min} \right)\zeta\left( t \right)+A_{min} (7)$$

The valve opening/closure relates to the pressure difference between upstream and downstream as follows:

| $\frac{d\zeta}{dt}=\left\{ \begin{aligned} \zeta K_{o}\Delta p, if P_{upstr.}\geq P_{downstr.} \\ \\ (1-\zeta)K_{c}\Delta p, if P_{upstr.}<P_{downstr.} \end{aligned} \right.$ | (8) |
| --- | --- |

With$K_{o}$ and $K_{c}$being the opening and closing constants of the valve.

Systemic Circulation

*1D Equations*

The arterial tree consists of a network of 103 arteries, including 55 main systemic arteries, the coronary circulation and a representation of the circle of Willis. Arteries are modelled as straight long tapered segments with viscoelastic walls. The 1D continuity and momentum equations are derived by integrating the Navier-Stokes equations:

$$\frac{\partial A}{\partial t}+\frac{\partial Q}{\partial x}+\psi=0 (9)$$

$$\frac{\partial Q}{\partial t}+\frac{\partial}{\partial x}\int_{A} u^{2}dA=-\frac{A}{\rho}\frac{\partial P}{\partial x}+2\pi R\frac{\mu}{\rho}\left. \frac{\partial\mu}{\partial r} \right|_{r=R}+Ab_{x} (10)$$

where A(x,t) is the instantaneous arterial lumen area of radius R(x,t), u(r,x,t) is the longitudinal velocity component, Q(x,t) is the volumetric flow rate, P(x,t) is the transmural pressure, b is the body force, and $\psi$ is the arterial wall seepage. Blood is assumed to be a Newtonian fluid with density ρ and dynamic viscosity μ. [E](https://journals.physiology.org/doi/full/10.1152/ajpheart.00037.2009#E1)quations 9 and 10 contain three primary variables (P, Q, and A), and thus one more equation is needed to close the system. This is given by the constitutive relation relating distending pressure, P, to local cross-sectional area, Α. The arterial lumen area A is assumed to be the sum of a nonlinear elastic $A^{e}$ and viscoelastic $A^{v}$ component:

$$A\left( t \right)=A^{e}\left[ P\left( t \right) \right]+A^{v}\left( t \right) (11)$$

The elastic component of the local area $A^{e}$is related to the instantaneous distending pressure, P, via the local area compliance $C_{A}^{e}$, which is divided into a location dependent term $C_{d}^{e}$ and a pressure dependent term $C_{p}^{e}$, such that:

$$C_{A}^{e}=a_{1}+\frac{b_{1}}{1+\left[ \frac{P-P_{maxC}}{P_{width}} \right]^{2}} (12)$$

With $a_{1},$ $b_{1},$ $P_{maxC}, P_{width}$ being constants. The location dependent compliance was estimated based on the Pulse Wave Velocity (PWV) of the measured segment:

$$C_{d}^{e}\left( \bar{d}, P_{ref} \right)=\frac{A}{\rho{PWV}^{2}\left( \bar{d}, P_{ref} \right)} (13)$$

The viscoelastic behavior as modelled by [7] is given by the convolution product between the elastic area, $A^{e},$and the derivative of a creep function, J(t).

$$A^{v}\left( t \right)=\int_{0}^{\infty} J(\tau)\cdot A^{e}\left[ P(t-\tau) \right]d\tau(14)$$

$$J\left( t \right)=\bar{a}\cdot\frac{e^{-\frac{t}{\tau_{2}}}-e^{-\frac{t}{\tau_{1}}}}{t} (15)$$

The time constants are $\tau_{1}$ and $\tau_{2}$. The alpha coefficient was determined to be $\bar{a}$ = 0.16-0:62$\bar{d}$. The value of $\bar{a}$was restricted to a minimum of 0.05. Considering the elastic and viscoelastic wall components, the continuity equation is rewritten as:

$$\frac{\partial P}{\partial t}+\frac{1}{C_{A}^{e}}\cdot\left( \frac{\partial Q}{\partial x}+\frac{\partial A^{v}}{\partial t} \right)=0 (16)$$

To calculate the shear stress term ($\tau_{w})$, Poiseuille flow was assumed:

$$u\left( r,t \right)=\frac{2}{\pi R^{2}}\left( 1-\frac{r^{2}}{R^{2}} \right)Q_{1} (17)$$

$$\tau_{w}\left( t \right)=-\frac{4\mu}{\pi R^{3}}Q_{1} (18)$$

*Terminal sites: 0-D models*

Peripheral arterial segments are terminated with a three-element Windkessel (WK3) model; this accounts for the cumulative effects of all distal vessels such as small arteries and capillaries beyond a terminal site. The WK3 model includes the proximal resistance ($R_{1}$), compliance ($C_{T}$), and distal resistance ($R_{2}$) of the vascular bed:

$$\frac{\partial Q}{\partial t}=\frac{1}{R_{1}}\frac{\partial P}{\partial t}+\frac{P}{R_{1}R_{2}C_{T}}-\left( 1+\frac{R_{1}}{R_{2}} \right)\frac{Q}{R_{1}C_{T}} (19)$$

Numerical Solution

The above equations were discretized according to a finite difference implicit scheme to solve for the pressure and flow waveforms over an entire heartbeat. An arbitrary pressure and flow are used to initialize the system. The system is iteratively solved using the Newton-Raphson method for 30 consecutive computational iterations. Cycle time was set to 0.8 s and time steps of 0.05 s were used to ensure proper convergence.


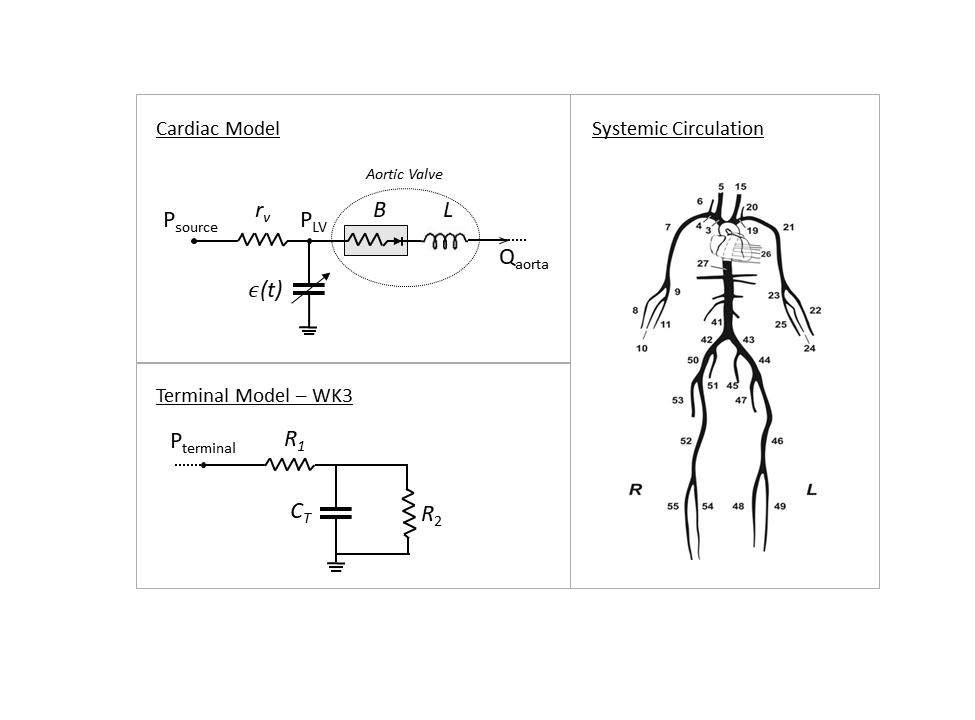


Figure S1.1. Schematic representation of the mathematical model of the cardiovascular system used in the study. Adapted from Reymond et al. [1].

**References**

1. Reymond P, Merenda F, Perren F, Rüfenacht D, Stergiopulos N. Validation of a one-dimensional model of the systemic arterial tree. Am J Physiol Heart Circ Physiol. 2009;297: H208-222. doi:10.1152/ajpheart.00037.2009

2. Suga H, Sagawa K. Instantaneous Pressure-Volume Relationships and Their Ratio in the Excised, Supported Canine Left Ventricle. Circ Res. 1974;35: 117–126. doi:10.1161/01.RES.35.1.117

3. Sagawa K, Suga H, Shoukas AA, Bakalar KM. End-systolic pressure/volume ratio: a new index of ventricular contractility. Am J Cardiol. 1977;40: 748–753.

4. Burkhoff D, Mirsky I, Suga H. Assessment of systolic and diastolic ventricular properties via pressure-volume analysis: a guide for clinical, translational, and basic researchers. Am J Physiol-Heart Circ Physiol. 2005;289: H501–H512. doi:10.1152/ajpheart.00138.2005

5. Stergiopulos N, Meister JJ, Westerhof N. Determinants of stroke volume and systolic and diastolic aortic pressure. Am J Physiol. 1996;270: H2050-2059. doi:10.1152/ajpheart.1996.270.6.H2050

6. Mynard JP, Davidson MR, Penny DJ, Smolich JJ. A simple, versatile valve model for use in lumped parameter and one-dimensional cardiovascular models. Int J Numer Methods Biomed Eng. 2012;28: 626–641. doi:10.1002/cnm.1466

7. Holenstein R, Niederer P, Anliker M. A viscoelastic model for use in predicting arterial pulse waves. J Biomech Eng. 1980;102: 318–325.
