# Supplementary material for: The effect of left ventricular contractility on arterial hemodynamics: A model-based investigation
Source: PLoS One. 2021 Aug 2;16(8):e0255561. doi: 10.1371/journal.pone.0255561 (PMC8328319; doi:10.1371/journal.pone.0255561)
Supplement: S2 Appendix — (DOCX) [file pone.0255561.s002.docx]

**S2 Appendix: Sensitivity Analysis**

A sensitivity analysis of the model results to preload and afterload is presented below.

**Sensitivity to preload**

Table S2.1 presents the results obtained after assuming that the high and low contractility simulations have the same LV EDP = 11 mmHg and EF = 54%. This was achieved by adapting the dead volume for the Ees=3 mmHg/mL scenario to 17mL. Note that the aortic pressure and flow, as well as the peripheral pressure are only minimally affected when preload is changed.

Table S2.1. Sensitivity of model predictions for aortic and peripheral pressure and flow curves to changes in preload.

| **Parameters** | **High Contractility Simulation**  **(Adopting the Frank-Starling mechanism)** | **High Contractility Simulation**  **(Assuming preload unchanged)** | **Low Contractility Simulation** |
| --- | --- | --- | --- |
| Preload (mmHg) | 8.2 | 11 | 11 |
| End-diastolic volume, EDV (mL) | 97 | 120 | 120 |
| Ejection fraction, EF | 68% | 54% | 54% |
| End-systolic elastance, Ees (mmHg/mL) | 3 | 3 | 1 |
| Dead volume, Vd (mL) | -2 | 17 | -60 |
|  |  |  |  |
| *Aortic flow* |  |  |  |
| Cardiac Output (L/min) (maintained) | 4.6 | 4.6 | 4.6 |
| Maximal Aortic Flow (mL/sec) | 453 | 452 | 359 |
| Timing of Maximal Aortic Flow (sec) (since beginning of ejection) | 0.06 | 0.06 | 0.07 |
| Maximal slope, dF/dt max (mL/sec^2^) | 1.53e4 | 1.45e4 | 1.09e4 |
|  |  |  |  |
| *LV P-V* |  |  |  |
| Maximal $P_{LV}$ (mmHg) | 114 | 115 | 120 |
| Timing of maximal $P_{LV}$ (sec)  (since beginning of ejection) | 0.14 | 0.14 | 0.23 |
| LV dP/dt max (mmHg/sec) | 14.9e2 | 14.4e2 | 8.7e2 |
|  |  |  |  |
| *Arterial pressure* |  |  |  |
| Aortic SBP (mmHg) | 113 | 112 | 120 |
| Aortic DBP (mmHg) | 67 | 68 | 68 |
| Aortic MAP (mmHg) | 91 | 93 | 92 |
| Aortic PP (mmHg) | 46 | 44 | 52 |
| Aortic AP (mmHg) | -1 | -2 | 9 |
| Aortic AIx (%) | -2.4 | -5.5 | 18.1 |
| Aortic dP/dt max (mmHg/sec) | 6.6e2 | 6.9e2 | 4.6e2 |
| Radial SBP (mmHg) | 148 | 144 | 135 |
| Radial DBP (mmHg) | 62 | 64 | 63 |
| Radial MAP (mmHg) | 87 | 88 | 88 |
| Radial PP (mmHg) | 85 | 80 | 72 |
| Radial AIx (%) | 47 | 50 | 77 |
| PP Amplification | 1.86 | 1.81 | 1.39 |
|  |  |  |  |
| *Wave Separation Analysis* |  |  |  |
| Forward pressure wave component amplitude (mmHg) | 28 | 28 | 29 |
| Forward pressure wave peak (mmHg) | 62 | 62 | 63 |
| Forward pressure wave peak timing (sec)  (since beginning of ejection) | 0.13 | 0.13 | 0.23 |
| Maximal slope forward dP/dt (mmHg/sec) | 6.6e2 | 6.4e2 | 4.4e2 |
| Backward pressure wave component amplitude (mmHg) | 21 | 21 | 23 |
| Backward pressure wave peak (mmHg) | 54 | 54 | 57 |
| Backward pressure wave peak timing (sec)  (since beginning of ejection) | 0.26 | 0.26 | 0.25 |
| Reflection coefficient (%) | 75 | 75.0 | 76.9 |

**Sensitivity to afterload**

Tables S2.2 and S2.3 contain the model results for the high and low contractility scenarios after scaling the model compliance and resistance by ±20%, respectively. Note that despite the changes in the absolute values of pressure and flow for the two scenarios, the major study conclusions still hold true: high Ees is linked with a higher peak flow, a lower aortic AIx and the Type C phenotype, higher pulse pressure amplification, and a steeper forward pressure wave.

Table S2.2. Sensitivity to total arterial compliance.

|  | **Compliance +20%** | | **Compliance -20%** | |
| --- | --- | --- | --- | --- |
| **Parameters** | **High Contractility Simulation** | **Low Contractility Simulation** | **High Contractility Simulation** | **Low Contractility Simulation** |
| Total arterial compliance (mL/mmHg) | 1.12 | 1.12 | 0.76 | 0.76 |
| End-diastolic volume, EDV (mL) | 97 | 120 | 97 | 120 |
| Ejection fraction, EF | 68% | 54% | 60% | 49% |
| End-systolic elastance, Ees (mmHg/mL) | 3 | 1 | 3 | 1 |
| Dead volume, Vd (mL) | -2 | -60 | -2 | -60 |
|  |  |  |  |  |
| *Aortic flow* |  |  |  |  |
| Cardiac Output (L/min) (maintained) | 4.6 | 4.6 | 4.4 | 4.4 |
| Maximal Aortic Flow (mL/sec) | 463 | 343 | 451 | 347 |
| Timing of Maximal Aortic Flow (sec) (since beginning of ejection) | 0.06 | 0.08 | 0.06 | 0.08 |
| Maximal slope, dF/dt max (mL/sec^2^) | 1.4e4 | 9.0^e^3 | 1.6e4 | 8.9e3 |
|  |  |  |  |  |
| *LV P-V* |  |  |  |  |
| Maximal $P_{LV}$ (mmHg) | 111 | 116 | 114 | 121 |
| Timing of maximal $P_{LV}$ (sec)  (since beginning of ejection) | 0.14 | 0.25 | 0.14 | 0.25 |
| LV dP/dt max (mmHg/sec) | 14.2e2 | 8.6e2 | 14.3e2 | 8.5e2 |
|  |  |  |  |  |
| *Arterial pressure* |  |  |  |  |
| Aortic SBP (mmHg) | 111 | 116 | 114 | 122 |
| Aortic DBP (mmHg) | 73 | 73 | 68 | 64 |
| Aortic MAP (mmHg) | 91 | 92 | 89 | 90 |
| Aortic PP (mmHg) | 38 | 43 | 46 | 58 |
| Aortic AP (mmHg) | -3 | 7 | -1.1 | 12 |
| Aortic AIx (%) | -9.1 | 16.3 | -2.4 | 20.7 |
| Aortic dP/dt max (mmHg/sec) | 6.1e2 | 3.9e2 | 7.1e2 | 4.5e2 |
| Radial SBP (mmHg) | 135 | 128 | 144 | 131 |
| Radial DBP (mmHg) | 67 | 68 | 60 | 61 |
| Radial MAP (mmHg) | 89 | 90 | 87 | 88 |
| Radial PP (mmHg) | 68 | 60 | 84 | 70 |
| Radial AIx (%) | 51.3 | 79.1 | 57.1 | 85.7 |
| PP Amplification | 1.79 | 1.39 | 1.82 | 1.21 |
|  |  |  |  |  |
| *Wave Separation Analysis* |  |  |  |  |
| Forward pressure wave component amplitude (mmHg) | 24 | 27 | 31 | 32 |
| Forward pressure wave peak (mmHg) | 59 | 62 | 62 | 64 |
| Forward pressure wave peak timing (sec)  (since beginning of ejection) | 0.13 | 0.24 | 0.13 | 0.26 |
| Maximal slope forward dP/dt (mmHg/sec) | 5.6e2 | 4.0e2 | 5.6e2 | 4.2e2 |
| Backward pressure wave component amplitude (mmHg) | 17 | 20 | 24 | 25 |
| Backward pressure wave peak (mmHg) | 52 | 55 | 55 | 57 |
| Backward pressure wave peak timing (sec)  (since beginning of ejection) | 0.25 | 0.27 | 0.24 | 0.26 |
| Reflection coefficient (%) | 71 | 74 | 77 | 78 |

Table S2.3. Sensitivity to peripheral resistance.

|  | **Resistance +20%** | | **Resistance -20%** | |
| --- | --- | --- | --- | --- |
| **Parameters** | **High Contractility Simulation** | **Low Contractility Simulation** | **High Contractility Simulation** | **Low Contractility Simulation** |
| Total peripheral resistance (mmHg*s/mL) | 1.31 | 1.31 | 0.96 | 0.96 |
| End-diastolic volume, EDV (mL) | 97 | 120 | 97 | 120 |
| Ejection fraction, EF | 59% | 48% | 70% | 56% |
| End-systolic elastance, Ees (mmHg/mL) | 3 | 1 | 3 | 1 |
| Dead volume, Vd (mL) | -2 | -60 | -2 | -60 |
|  |  |  |  |  |
| *Aortic flow* |  |  |  |  |
| Cardiac Output (L/min) (maintained) | 4.3 | 4.3 | 5.1 | 5.1 |
| Maximal Aortic Flow (mL/sec) | 451 | 296 | 528 | 389 |
| Timing of Maximal Aortic Flow (sec) (since beginning of ejection) | 0.06 | 0.08 | 0.06 | 0.08 |
| Maximal slope, dF/dt max (mL/sec^2^) | 1.3^e^4 | 8.6e3 | 1.6^e^4 | 1.1e4 |
|  |  |  |  |  |
| *LV P-V* |  |  |  |  |
| Maximal $P_{LV}$ (mmHg) | 114 | 126 | 95 | 109 |
| Timing of maximal $P_{LV}$ (sec)  (since beginning of ejection) | 0.15 | 0.23 | 0.16 | 0.25 |
| LV dP/dt max (mmHg/sec) | 14.3e2 | 8.5e2 | 14.1e2 | 8.5e2 |
|  |  |  |  |  |
| *Arterial pressure* |  |  |  |  |
| Aortic SBP (mmHg) | 114 | 126 | 95 | 108 |
| Aortic DBP (mmHg) | 67 | 79 | 56 | 62 |
| Aortic MAP (mmHg) | 94 | 98 | 75 | 81 |
| Aortic PP (mmHg) | 47 | 47 | 39 | 46 |
| Aortic AP (mmHg) | -2 | 14 | -5 | 4 |
| Aortic AIx (%) | -3.8 | 29.2 | -12.8 | 8.9 |
| Aortic dP/dt max (mmHg/sec) | 6.5e2 | 4.3e2 | 6.4e2 | 5.0e2 |
| Radial SBP (mmHg) | 140 | 134 | 124 | 124 |
| Radial DBP (mmHg) | 66 | 74 | 53 | 56 |
| Radial MAP (mmHg) | 92 | 96 | 73 | 79 |
| Radial PP (mmHg) | 74 | 60 | 71 | 68 |
| Radial AIx (%) | 56.7 | 89.7 | 40.9 | 78.4 |
| PP Amplification | 1.57 | 1.28 | 1.82 | 1.48 |
|  |  |  |  |  |
| *Wave Separation Analysis* |  |  |  |  |
| Forward pressure wave component amplitude (mmHg) | 28 | 29 | 24 | 29 |
| Forward pressure wave peak (mmHg) | 61 | 68 | 51 | 58 |
| Forward pressure wave peak timing (sec)  (since beginning of ejection) | 0.15 | 0.21 | 0.17 | 0.24 |
| Maximal slope forward dP/dt (mmHg/sec) | 6.3e2 | 4.9e2 | 5.8e2 | 5.1e2 |
| Backward pressure wave component amplitude (mmHg) | 21 | 21 | 18 | 22 |
| Backward pressure wave peak (mmHg) | 55 | 59 | 45 | 51 |
| Backward pressure wave peak timing (sec)  (since beginning of ejection) | 0.25 | 0.25 | 0.24 | 0.25 |
| Reflection coefficient (%) | 75.0 | 72.4 | 75.0 | 75.8 |
